# Hemorrhage Detection Based on 3D CNN Deep Learning Framework and Feature Fusion for Evaluating Retinal Abnormality in Diabetic Patients

**DOI:** 10.3390/s21113865

**Published:** 2021-06-03

**Authors:** Sarmad Maqsood, Robertas Damaševičius, Rytis Maskeliūnas

**Affiliations:** 1 Department of Software Engineering, Kaunas University of Technology, 51368 Kaunas, Lithuania; sarmad.maqsood@ktu.edu; 2 Department of Applied Informatics, Vytautas Magnus University, 44404 Kaunas, Lithuania; rytis.maskeliunas@vdu.lt; 3 Faculty of Applied Mathematics, Silesian University of Technology, 44-100 Gliwice, Poland

**Keywords:** medical image processing, hemorrhage detection, retinal fundus image, diabetic retinopathy, feature fusion, deep learning

## Abstract

Diabetic retinopathy (DR) is the main cause of blindness in diabetic patients. Early and accurate diagnosis can improve the analysis and prognosis of the disease. One of the earliest symptoms of DR are the hemorrhages in the retina. Therefore, we propose a new method for accurate hemorrhage detection from the retinal fundus images. First, the proposed method uses the modified contrast enhancement method to improve the edge details from the input retinal fundus images. In the second stage, a new convolutional neural network (CNN) architecture is proposed to detect hemorrhages. A modified pre-trained CNN model is used to extract features from the detected hemorrhages. In the third stage, all extracted feature vectors are fused using the convolutional sparse image decomposition method, and finally, the best features are selected by using the multi-logistic regression controlled entropy variance approach. The proposed method is evaluated on 1509 images from HRF, DRIVE, STARE, MESSIDOR, DIARETDB0, and DIARETDB1 databases and achieves the average accuracy of 97.71%, which is superior to the previous works. Moreover, the proposed hemorrhage detection system attains better performance, in terms of visual quality and quantitative analysis with high accuracy, in comparison with the state-of-the-art methods.

## 1. Introduction

Diabetic Retinopathy (DR) is the major cause of vision impairment and blindness in the developed nations of age between 21 and 77 years [1,2]. The World Health Organization (WHO) predicted that in 2030 around 300 million people will suffer from diabetes [3]. The main cause of diabetes is when the pancreas fails or is not fully able to secrete enough insulin. The persons, who are suffering from diabetes for a longer period of time, have their retina slowly degenerated [4]. As it progresses, the patient’s vision starts deteriorating leading to DR. Ophthalmoscopy, fundus photography, or a dilated fundus exam is used to evaluate the consequences of nature and status of effect on the eyes due to diabetes. The long-term effects of diabetes are the rapture, leakage, and damage to blood vessels in the retina, which increases the amount of glucose in the blood and will cause a typical pathology known as DR [5,6]. Blindness due to DR can be reduced if it is diagnosed in the early stages by proper screening. However, the monitoring of DR is executed manually which is time consuming. The automated screening of DR can overcome the manual screening that can filter out healthy obvious samples and indicates only suspected cases to ophthalmologists [7,8].

DR is an eye condition associated with complications of diabetes mellitus [9], which damages the retinal blood vessel. The automated DR screening results from retinal fundus images consists of red lesions, micro-aneurysms (MAs), hemorrhages, and bright lesions exudates [10]. When the lipids and proteins are leaked from the vessel it makes yellow spots on the retina known as exudate. Exudates have two categories, i.e., hard and soft exudates, also known as cotton wool spots (CWS) [11]. Hard exudates are visible as a yellowish with finite edges, sharply defined and appearing shiny in imaging, individually and collectively. Soft exudates are visible as a whitish with indistinct edges, giving the feel of diffuse cotton shape. Soft exudates appear due to blockage of nerve fibers that receive blood supply from the retinal arteries so that the axon’s nerve fibers become enlarged [12]. DR is known to have particular symptoms covering MAs, hemorrhages, soft exudates or CWS, hard exudates, neovascularization (NV), and macular edema (ME) [13,14].

Currently, there are numerous DR screening approaches for the diagnosis, i.e., color fundus photography and fundus fluorescein angiograms (FFA) to determine pathological signs [15]. Color fundus photography method is economical and can save the data easily, therefore it is more appropriate by ophthalmologists for DR screening. Contrastingly, FFA differentiate better between the MAs and hemorrhages, due to its invasiveness, cost, and risk of allergic reactions, fundus imaging is the desired method [16]. The DR screening procedure performed by the ophthalmologists is time consuming, therefore it is deemed necessary to employ computer-based technology for the automatic detection and analysis of DR and pathological signs in the color fundus images to make the diagnosis more accurate and more accessible to people in the remote communities [17,18] as a part of remote health (telehealth) infrastructure services [19].

**Figure 1:**
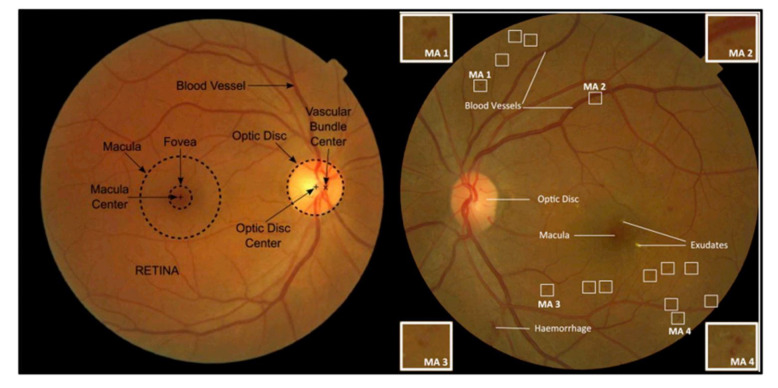
Colored fundus image marked with important retinal features [12]. Reprinted from ref [12]. Copyright 2018 Elsevier.

Hemorrhages are the early stage lesions of DR. Hemorrhages are placed in the deep middle layer of the retina and are usually round dark red spots, flamboyant spots, bright red, linear, and long strip [20]. Hemorrhages normally coexist with MAs, and the rate of clinical DR is categorized according to the existence and number of these two lesions. Hence, the accurate hemorrhage detection is essential for the automatic detection and effectual analysis of DR [21]. Figure 1 illustrates the retinal fundus image marked with numerous features like blood vessels, optic disc, fovea, macula, hemorrhages, exudates, etc. Hemorrhage detection is very challenging for early diagnosis of DR because of the variation in color, size, texture, shape, and also contains similar color contrast with its background. The detection of hemorrhages is the most challenging task in fundus image analysis.

The expert-based DR detection process is time consuming, costly, manual, and requires extra efforts to run the equipment. The accurate and automatic hemorrhage detection is never easy in terms of image processing and exhibits several limitations due to the following reasons [22].

1. Hemorrhages occur in places where the contrast is significantly poor.
2. False hazard because of the existence of blood vessels.
3. Detection performance may vary by disparate sizes of MAs and hemorrhages.
4. Existing DR screening methods are computationally complex and take a longer processing time to detect the accurate hemorrhages.

Hence, the accurate automatic hemorrhage detection method is needed to take care of the patients. Regular retinal imaging over a time interval has quickly become the standard of care for a variety of eye diseases such as glaucoma, diabetes, hypertensive retinopathy [23], and macular degeneration. Today, computerized diagnostic systems based on image processing are becoming increasingly popular to make it easier for doctors and shorten the time of diagnosis [24,25].

To this end, we propose a novel method for the automatic detection of early pathological signs of DR in diabetic patients namely hemorrhages after the development of MAs, having the following contributions:

1. A modified Contrast Limited Adaptive Histogram Equalization (CLAHE) method is used as a preprocessing step to enhance the edge details from the input source images.
2. A novel 3D Convolutional Neural Network (CNN) model for the accurate segmentation of hemorrhages from the retinal images with high accuracy and early detection.
3. A modified pre-trained VGG19 deep learning architecture is used for feature extraction, and it performs transfer learning to retrieve the selected datasets.

The proposed algorithm reduces the time it takes for an ophthalmologist to diagnose hemorrhages, while ensuring reliable detection accuracy. The proposed automatic system is cost effective and presents accurate results with less processing time.

The remaining paper is structured as follows. Section 2 reviews the prominent related work on hemorrhage detection and classification. In Section 3, the detailed methodology of the proposed method is discussed. Section 4 analyses the performance of the proposed method in comparison with other state-of-the-art methods and, finally, Section 5 concludes this paper with future research goals.

## 2. Related Work

Modern healthcare methods actively use retinal fundus images for the diagnosis [26,27,28,29]. In this section, we critically review the prominent work on hemorrhage detection from the retinal fundus images.

Many researchers have worked on the automated detection and classification of the hemorrhages using retinal fundus images. For example, Tang et al. [20] developed a method based on splat feature classification to detect the hemorrhages in retinal fundus images using supervised learning. This method uses the MESSIDOR database and attains the receiver operating characteristic curve of 0.96. Srivastava et al. [21] presented a frangi filter to recognize red lesions and blood vessels. These methods can be used on different scales of patches of different sizes. Each grid is designed with a kernel and multiple cores with SVM are used to diagnose lesions of different sizes. The proposed method used 143 images for MAs and hemorrhage detection and obtained the receiver operating characteristic curve of 0.97 and 0.92, respectively.

Seoud et al. [30] proposed an algorithm for MAs and hemorrhage detection using color retinal fundus images using dynamic shape features. These features reflect the evolution of shape during image flooding and can distinguish lesions and vascular segments. This approach uses the MESSIDOR database and obtained the FROC score and ROC curve of 0.420 and 0.899, respectively. Wu et al. [31] presented an automatic hemorrhage detection method based on two dimensional gaussian fitting. The image is enhanced using contrast enhancement and watershed segmentation is used to extract the hemorrhages. The two-dimensional Gaussian adaptation is used to extract visual characteristics from a hemorrhages candidate. This method used the DIARETDB1 database using 219 retinal fundus images and obtained the sensitivity, specificity and accuracy of 100%, 82%, and 95.42%, respectively.

Mumtaz et al. [32] proposed an automatic hemorrhage detection approach. The noise is removed through image enhancement and normalization. The blood vessels are segmented from hemorrhages using scale-based methods and finally by using the gamma correction and thresholding method the hemorrhages are detected. This algorithm used the DIARETDB1 database and attained a specificity, sensitivity, and accuracy of 84%, 87%, and 89%, respectively.

Tan et al. [33] developed an approach which automatically detects the exudates and hemorrhages using convolutional neural networks (CNNs). CLEOPATRA database is used and has a sensitivity of 0.6257.

Prasad et al. [34] presented a hemorrhage detection method using retinal fundus images through feature classification by extracting the features using filter bank outputs and applied gaussian filters on green channel result. Then using the wrapper and filter approach the final set of extracted features are determined.

Orujov et al. [35] suggested a contour detection based method, which uses Mamdani (Type-2) fuzzy rules for blood vessel detection in retinal fundus images. The approach has achieved an accuracy of 0.865, 0.939, 0.950 for the STARE, DRIVE and ChaseDB datasets, respectively.

Shankar et al. [36] proposed an automated detection of DR using a deep learning approach. The segmentation based on histogram is used to extract the features and a synergic deep learning method is used to classify the fundus DR images. This method uses the MESSIDOR database.

Gadekallu et al. [37] proposed an automated detection algorithm by employing principal component analysis firefly-based deep learning approach to extract the important features from the retinal fundus image.

Kumar et al. [38] presented an enhanced technique to detect the hemorrhages using fundus images. The segmentation method based on watershed transform is used to segment out the candidate region and radial function neural network is used for the classification. This method attained a sensitivity and specificity of 87%, 93% respectively.

Joshi et al. [39] proposed a method for the recognition of hemorrhages based on morphological segmentation and geometrical feature approaches. This method uses the DIARETDB1 dataset and attains an accuracy of 95.47%.

Qureshi et al. [40] presented the hemorrhage detection method using a multi-layer framework of neural networks. The convolutional neural network is used to extract the features to obtain the candidate region. This method attained the average sensitivity of 92.20%, specificity of 95.10%, and classification accuracy of 98%.

Bae et al. [41] proposed an algorithm based on normalized cross-correlation template matching for the detection of hemorrhages. This method attained a sensitivity of 85%. Sirajudeen et al. [42] used the multi-scale local binary pattern to obtain the features and support vector machine to recognize the hemorrhages.

From the literature review, we can conclude that there are still various concerns related to information extraction to DR detection that need serious attention, such as (i) red lesions occur in places where the contrast is significantly poor, (ii) false hazard because of the existence of blood vessels, and (iii) detection performance may vary by disparate sizes of MAs and hemorrhages.

To resolve these aforementioned problems, we propose a novel hemorrhage detection algorithm that is elaborated on in the following section.

## 3. Proposed Hemorrhage Detection Technique

**Figure 2:**
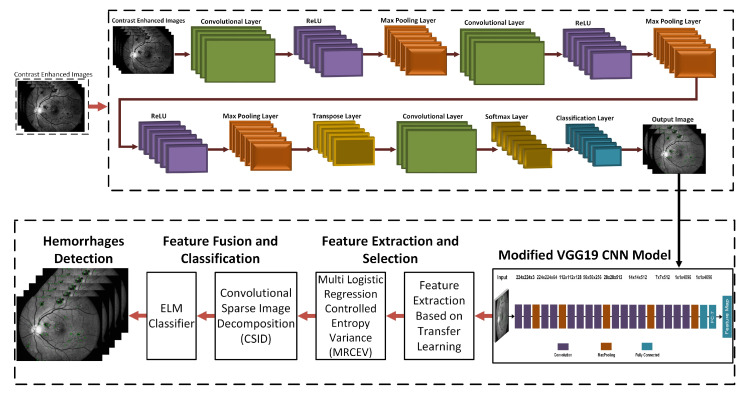
Schematic diagram of the proposed 3D-CNN selection of feature for hemorrhage detection and classification.

This section shows our proposed novel approach for hemorrhage detection and classification. The proposed method comprises eight phases that include green channel extraction, contrast enhancement, 3D CNN based segmentation, training models, deep learning features, feature extraction using transfer learning, feature selection, and feature fusion and classification as displayed in the schematic model in Figure 2. These steps are detailed in the following subsections.

### 3.1. Green Channel Extraction

Colored retinal fundus images are in imperfect contrast. Therefore, it is very important to refine the contrast of the images. To find our region of interest (ROI) the color images are converted into the green channel. The reason for using the green plane is due to the highest contrast between hemorrhages, blood vessels, optic disc, exudates, and the background as compared to the blue and red plane. In addition, the red lesions (hemorrhages) and blood vessels appear dark and the white lesions (exudates) and optic disc appear bright in the green plane image. Retinal fundus images need to be separated into three channels and we use only one of them.

**Figure 3:**
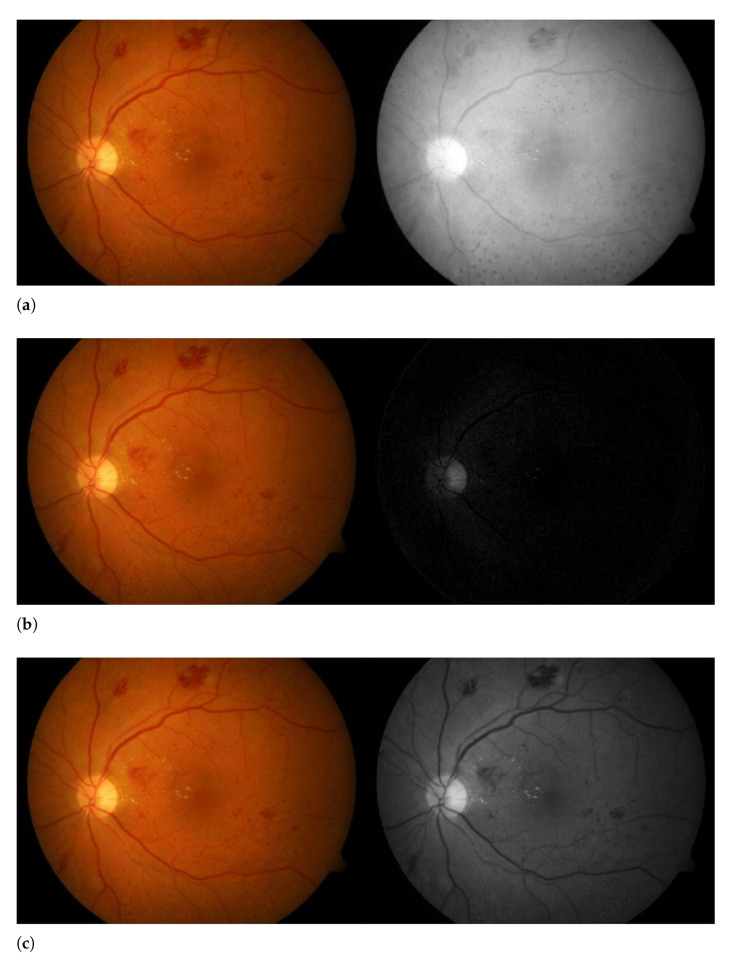
Left: the retinal fundus image. Right: (**a**) Red channel extraction, (**b**) Blue channel extraction, (**c**) Green channel extraction.

The extraction of the red, blue, and green channels of the retinal fundus image is shown in Figure 3. As displayed in Figure 3a,b it can be observed that the red and blue channel is not extracting the complete information. The blue channel extracted from the retinal image has poor contrast and does not contain all the necessary information for further processing. In the red channel, the vessels in the fundus images are found to be noticeable, on the other hand, the red channel incorporates much noise or sometimes it is just saturated. In Figure 3c, the green channel provides full detailed information of the retinal fundus image. Green channel extraction from the color retinal images provides a prominent outcome in the contrast of blood vessels as in this channel it darkens the blood vessels on a bright background. So, in this paper, we have used the green channel for the detection of hemorrhages.

### 3.2. Contrast Enhancement

Contrast enhancement is a main pre-processing step for diagnosis processes [43]. The source retinal fundus image has poor contrast due to inadequate illumination. To enhance the low contrast images the histogram equalization approach seems to be a more effective technique. A modified CLAHE [43] is used to refine the contrast and keep the average brightness of the input image. CLAHE affects small segments of the image (called tiles). The contrast of each mosaic is enhanced, rather than the entire image in retinal images, so the histogram in the output area roughly matches the specified histogram. After leveling, adjacent tiles are joined using linear interpolation to remove the artificial boundary. CLAHE uses a user-defined clipping threshold, which is used to limit the enhancement when clipping the histogram. The crop level reduces the noise level, and the crop level also sets the contrast level to improve the histogram. In this paper, we used 0 to 0.01.

Firstly, the source image is divided into non overlapping related regions. The total number of image tiles is equal to *M* × *N*. The histogram of each non overlap related region is computed to gray levels that exist in the image array. Equation (1) computed the contrast limited histogram of the non-overlapping related part by clip limit as:

(1) $$I_{avg}=\frac{N_{x}\times N_{y}}{N_{gray}},$$

where $I_{avg}$ is the pixel average number, $N_{gray}$ is the number of gray levels in the non overlapping related part, $N_{x}$ and $N_{y}$ is the number of pixels in the non-overlapping area dimensions *x* and *y*. The clip limit is calculated in Equation (2) as:

(2) $$I_{CL}=N_{clip}\times I_{avg},$$

where $I_{CL}$ is the clip limit, $N_{clip}$ is the normalized clip limit of range between [0, 1]. The pixels are clipped when the number of pixels is greater than $I_{CL}$. The remaining average pixels is distributed to each gray level as:

(3) $$I_{avg,gray}=\frac{N_{wc}}{N_{gray}},$$

where $N_{wc}$ represents the whole number of clipped pixels. Move the remaining pixels until all remaining pixels are linked. The pixel redistribution step is calculated in Equation (4) as:

(4) $$I_{step}=\frac{N_{gray}}{N_{cr}},$$

where $N_{cr}$ is the number of truncated pixels remaining. Furthermore, by using the Rayleigh transform in each region the intensities values are refined in Equation (5) as:

(5) $$I_{y}=I_{min}+\sqrt{2\alpha^{2}ln\left(\frac{1}{1-P_{in}}\right)I_{step}},$$

where $P_{in}$ is the cumulative probability which is used to develop transfer function, $I_{min}$ is represents the lower bound of pixel values and α is the scaling parameter. The output probability density of each intensity value is given in Equation (6) as:

(6) $$I_{rox}=\frac{\left(I_{y}-I_{min}\right)}{\alpha^{2}}.exp\left(-\frac{{\left(I_{y}-I_{min}\right)}^{2}}{2\alpha^{2}}\right)forI_{y}\geq I_{min}.$$

Greater value of α shows more notable contrast enhancement in an image, however it will increase saturation value and amplify the noise levels. By rearranging the output of the obtained transfer function using linear contrast stretching, the effect of sudden changes can be suppressed. The linear contrast stretching can be expressed in Equation (7) as:

(7) $$I_{i}=\frac{I_{rox}-w_{min}}{w_{max}-w_{min}},$$

where $I_{rox}$ is the obtained transfer function, $w_{max}$ and $w_{min}$ represents the maximum and minimum transfer function value. $I_{i}$ is employed to green channel extraction images to obtain the contrast enhanced images. Contrast enhancement results in improved edges in the input images.

**Figure 4:**
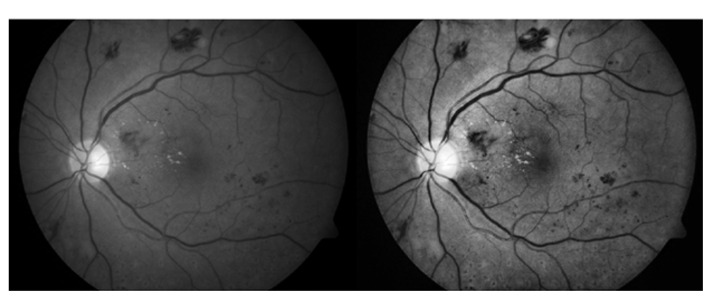
The retinal fundus image for contrast enhancement. Left: Green channel extraction, Right: Final contrast enhancement.

Figure 4 illustrates the contrast enhancement from the green channel. From the images, it can be observed that after applying our modified contrast enhancement method, the image gradients are greatly enhanced. On completion of this phase, the proposed method enters the third stage, which is elaborated in the below subsection.

### 3.3. 3D CNN Based Segmentation Model

**Figure 5:**
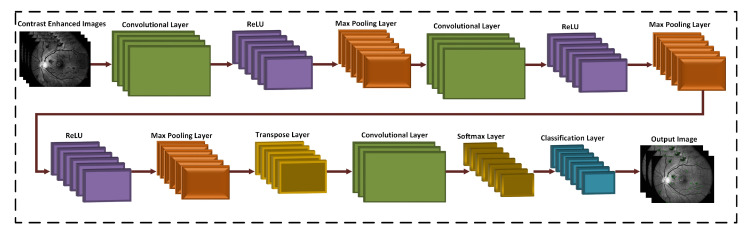
Architecture of the proposed 3D-CNN for hemorrhage extraction.

The 3D CNN based framework is proposed for the hemorrhage detection. This architecture deals with 3D images for the calculation of features, while passing the input to other layers in the form of multiple corrections. The architecture of the proposed 3D CNN is displayed in Figure 5. We take a source image I(x,y) having dimensions *M* × *N* × *P* where *M* = 512, *N* = 512, and *P* = 3, respectively. *N*, *M*, and *P* represents the row pixels, column pixel values, and the number of channels which are 3 in this study, respectively. Given that ξ represents a color block of size 32 × 32 × 3 and *V* denotes the *i*-th color block, the convolutional layer is expressed in Equation (8) as:

(8) $$\xi_{i}^{q}=I_{i}\left[\sum_{m=1}^{\kappa -1}\chi_{i}^{m,n}\times \xi_{i-1}^{q}+\vartheta_{i}^{q}\right],$$

where $\xi_{i}^{q}$ represents the current layer, $\chi_{i}^{m,n}$ denotes the weighted matrix, $\xi_{i-1}^{q}$ represents the precursory layer, and $\vartheta_{i}^{q}$ is each patches bias value. The hidden layer of each weighted matrix $\chi_{i}^{m,n}$ is learned and returns a matrix for the 4D kernel. The kernels are linked together in 4D as:

(9) $$\left[\xi_{i}^{m,1},\xi_{i}^{m,2},\xi_{i}^{m,3},...,\xi_{i}^{m,\kappa -1}\right].$$

After the convolutional layer the ReLu activation feature is used to quickly perform the training. This function returns the identities of all positive values and zeros for all negative characteristics. The following expression in Equation (10) provides a more complex image model that will be used later to better determine the nature of the pixel.

(10) $$\xi_{i-1}=\xi_{i-1}^{1},\xi_{i-1}^{2},\xi_{i-1}^{3},...,\xi_{i-1}^{\kappa -1}.$$

In addition, this function helps eliminate the overfitting problem which is mathematically computed in Equation (11) as:

(11) T=max(0,ξ).

**Figure 6:**
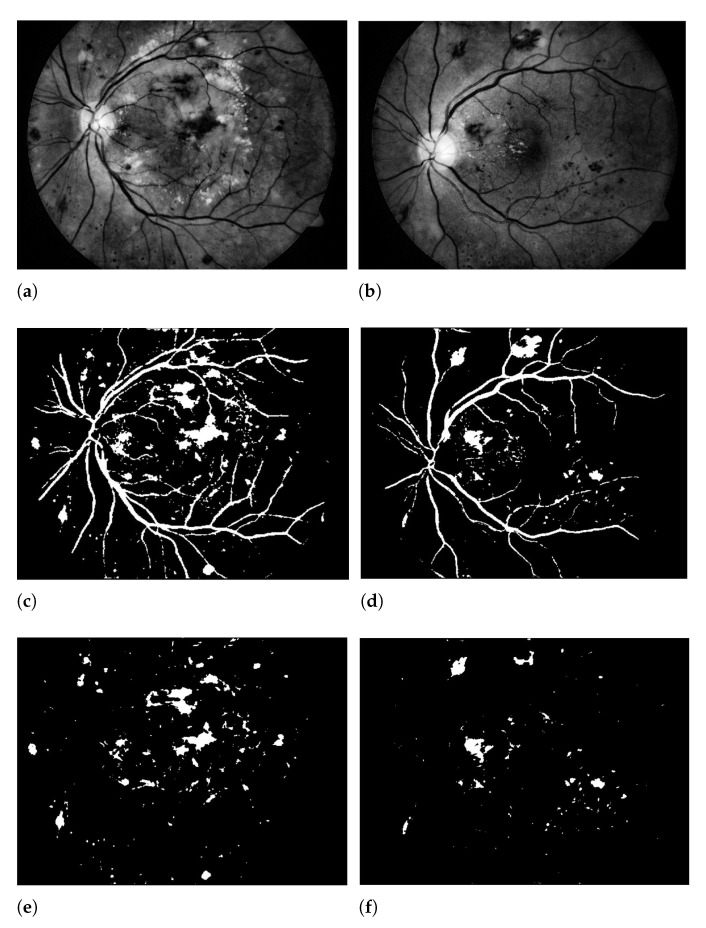

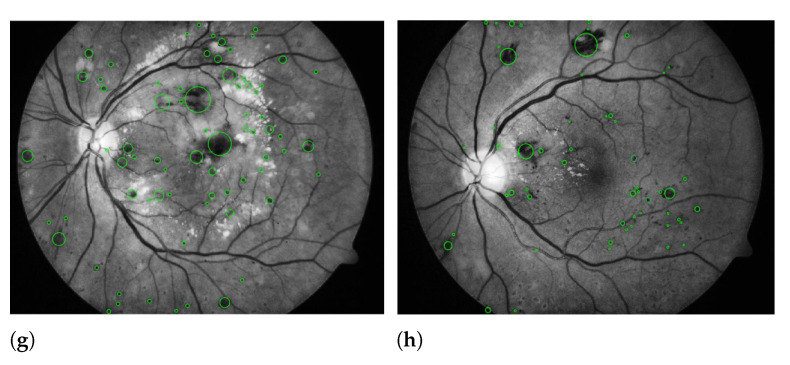
The retinal fundus image. (**a**,**b**) Proposed modification to contrast enhanced images, (**c**,**d**) Background estimated results using OTSU method corresponding to the 1st row, (**e**,**f**) Proposed 3D-CNN segmentation results corresponding to the 1st row, (**g**,**h**) Extraction of hemorrhages corresponding to the 1st row.

Afterwards, a max-pooling layer is down sampled in CNN layers that reduces the spatial size of the feature map. In our proposed work, two max-pooling layers are created to reduce the features dimension and remove redundant spatial information as displayed in Figure 6.

Like other interpolation methods, i.e., bicubic, bilinear, nearest neighbor, etc., the transposed convolution layer is used for the upsampling. This layer contains numerous parameters which learned and helped to create a new image. Finally, add a pixel label classification layer to segment the hemorrhages according to the cross-entropy function shown in Equation (12).

(12) $$\psi \left(\xi ,Q\right)=-\frac{1}{V}\sum_{i=1}^{V}ln\left(R_{Q}\right),$$

where ξ represents the dimension patches of 32 × 32 × 3, *C* represents the complementary true labels, *V* denotes the i-th patches of an image, and $R_{Q}$ represents the hind probabilities for actual class *Q*. On completion of this stage, the proposed method enters the fourth stage, which is elaborated in the below subsection.

### 3.4. Training Models

The input layer of patch 32 × 32 × 3 is selected with center normalization of 0. The first layer of convolution is created, stride is [1 1] and padding is [1 1 1 1]. Afterwards, the max pooling layer of 2 × 2 added of stride [1 1] and padding [0 0 0 0]. Then a second convolutional layer is selected of stride [1 1] and padding [1 1 1 1]. The second max pooling layer is created of stride [2 2] and padding [0 0 0 0]. The detailed description of all the layers are displayed in Table 1, where the neural network (NN) is trained. To train the NN, activate the parameters, such as the sigmoid activation function. The minimum batch size is 64, the learning rate is 0.001, the number of epochs is 100, and a total of 500 iterations are executed. A ReLu activation function is employed after each networks layer except the last layer where a sigmoid activation function is employed. The Sigmoid function Sι is mathematically expressed in Equation (13) and in Equation (14) as:

(13) $$S=\sum_{i=1}\pi_{i}+\eta_{i}\chi_{i}.$$

(14) $$S_{\iota }=\frac{1}{1+e^{-S}}$$

The trained CNN is registered as a new network and used in the testing method. The final achieved results are then improved using morphological operations (opening and closing). Figure displayed the obtained results of segmentation by using our proposed technique.

On completion of this phase, the proposed method enters the fifth stage, which is elaborated in the below subsection.

### 3.5. Deep Learning Features

In this work, the deep learning features were obtained using the pre-trained CNN-model VGG19 [44]. The VGG19 model is also trained on ImageNet dataset. The motivation behind choosing this model is that the VGG19 network has learned rich feature representations for a wide range of images and the VGG19 model has achieved significant performance in the image competition. The modified VGG19 contains 16 layers of convolution, 19 layers of learnable weights, 3 fully connected layers, and output layer, which are used for the transfer learning. The size of the source image for the modified model is 224 × 224 × 3. The first convolutional layers are 1 × 1 × 64 and 3 × 2 × 3 × 64 for the bias and learnable weights. For the first convolution layer, the total learnable weight is 1792 and for second the learnable weight are 36,928. This layer extracts local features from the image.

(15) $$H_{\iota }=S_{\iota }+\sum_{n=1}^{M-1}\eta_{\iota ,n}\times \varphi_{m}^{M-1},$$

where $H_{\iota }$ represents the output layer. $S_{\iota }$ denotes the bias value, $\eta_{\iota ,n}$ denotes the *k*-th feature value of map filter, and $\varphi_{m}$ is the output layer of M−1.

The weights and the bias that can be learned from the first fully connected layer are: 4096 × 25,088 and 4096 × 1. A dropout layer is created among the fully connected layers, and the compression ratio is 50%. The total number of learnable features in fully connected layers 7 is 16,782,313, and the weights that can be learned are 4096 × 4096. In the final fully connected layer, the total learnable numbers are 4,097,000 and the learning weight is 1000×4096. Therefore, when activated, a feature map vector of size 1 × 1 × 1000 is returned. The perfect combination of layer 1 and layer 2 results in a map vector size of 1 × 1 × 4096.

On completion of this phase, the proposed method enters the sixth stage, which is elaborated in the below subsection.

### 3.6. Feature Extraction Using Transfer Learning

**Figure 7:**
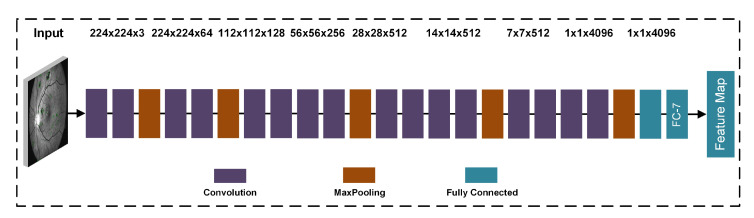
The modified VGG19 architecture for the features extraction.

The transfer learning based feature extraction is used to retrain the modified VGG19 based CNN model on our datasets. The modification to the VGG19 architecture is displayed in Figure 7. The input and output convolutional layers are determined as feature mapping. The 55:45 (training:testing) strategy is used with labeled data. The first layer of convolution is selected as input, and fully connected layer 7 as output. After completing the activation of CNN, we acquired the training and testing vectors. The training and testing vector are used in the next process of feature fusion. The final feature vector is achieved with the size 1 × 4096 on the fully connected 7 feature layer.

On completion of this phase, the proposed method enters the seventh stage, which is elaborated in the below subsection.

### 3.7. Feature Selection

The feature selection is used to achieve improvement in the accuracy of classification, eliminate the redundancy between features and pass only robust features for accurate classification, and help us to reduce the number of predictions and complete the testing process faster. The Multi Logistic Regression Controlled Entropy Variance (MRCEV) [45] approach is used for feature selection. The partially derived based activation function is utilized to remove inconsequential properties and transfer the remaining trusted properties to the entropy distribution function. This will be a new vector with only positive values. The mathematical expression is computed in Equation (15) and in Equation (17) as:

(16) $$\ell_{i}=\sum_{k=1}^{n}\partial_{i,k}\varrho_{k},$$

(17) $$s\left(\tau |\upsilon \right)=\frac{exp(\ell_{i})}{\sum_{k=1}^{l}\partial_{i,k}exp\left(\ell_{i}\right)},$$

where τ represents the corresponding labels and τϵ*R* and υ is the probability of *i*-th class. The regression parameter $\ell_{i}$ = $\ell_{0}$,$\ell_{i1}$,$\ell_{2}$,...,$\ell_{n-1}$ is acquired by reducing the possibility of negative properties.

If the features are independent, the polynomial distribution is calculated as:

(18) $$R_{\Psi }=-\sum_{j}^{m}\sum_{i=1}^{n}\tau_{i}lns\left(\tau |\upsilon \right),$$

(19) $$T\left(ℑ\right)=R_{\Psi }+\partial R_{⋄},$$

(20) $$R_{⋄}=\sum_{j=1}^{k}\left|\ell_{i}\right|,$$

where $\ell_{i}$ is the regularization parameter which is connected to obtain the sparse model and $R_{⋄}$ denotes the function called entropy variance.

We pass the selected features to this function to clearly distinguish all features for classification. On completion of this phase, the proposed method enters the final and eighth phase, which is elaborated in the following subsection.

### 3.8. Feature Fusion and Classification

This is the hot topic in the application area of pattern recognition. Finally, the Convolutional Sparse Image Decomposition (CSID) fusion method [46] is used to concatenate the feature vectors selected in the matrix to acquire a feature vector for the classification. The final fusion is computed in Equation (21) as:

(21) $$\varsigma_{r,s}=min_{\zeta_{r,s}}\frac{1}{2}∥R_{⋄}-\sum_{s=1}^{S_{q}}h_{q,s}*\zeta_{q,s}-\sum_{u=1}^{U_{r}}h_{r,s}*\zeta_{r,s}{∥}_{2}^{2}+\lambda_{r}\sum_{s=1}^{S_{t}}\left|\right|\zeta_{q,r}{\left|\right|}_{1}.$$

Continue this process until all pairs have been compared. $\varsigma_{r,s}$ is the final fused vector. This step is time consuming but our main aim is to improve the accuracy. The fused vector is further used for the final classification using an extreme learning machine (ELM) [47]. The formulation of ELM is computed in Equation (22) as:

(22) $$\sum_{j=1}^{V}\Upsilon_{j}\Pi_{j}\left(\chi_{j}\right)=\sum_{i=1}^{V}\Upsilon_{j}\Pi \left(\chi_{j}\chi_{(}k\right)+W_{\nu }),$$

where *V* represents the hidden layers, $\Upsilon_{j}$ represents weighted output vector, $\chi_{j}$ represents the weighted input vector, and $W_{\nu }$ represents the offset value. We further minimize the function to enhance the stability of ELM as computed in Equation (23):

(23) $$min\frac{1}{2}\Upsilon_{j}+\frac{1}{2}x\sum_{j=1}^{M}\left|\right|\phi_{j}{\left|\right|}^{2},s.t.\Upsilon^{y}=0,\Upsilon^{y}\left(\chi_{j}\right)=u_{j}-\phi_{j},$$

where *x* represents the penalty parameter, $\phi_{j}$ represents the errors in training, and $u_{j}$ represents samples corresponding labels.

## 4. Performance Evaluation

### 4.1. Environment and Datasets

The proposed method is compared with some of the other techniques to clarify the efficacy and the perfection of the algorithm. The experiments are executed on a laptop with a Intel(R) Core(TM) i7−9750H2.6 GHz processor with 12 GB RAM. All models are developed and experiments are implemented in TensorFlow v1.12 and trained on the NVIDIA GeForce GTX 1650 GPU.

To evaluate the performance of our proposed system for hemorrhage detection, we used High Resolution Fundus Image (HRF) [48], Digital Retinal Images for Vessel Extraction (DRIVE) [49], STructured Analysis of the Retina (STARE) [50], MESSIDOR [51], DIARETDB0 [52], and DIARETDB1 [53] databases. In this experiment, we used total of 1509 color fundus images, in which HRF dataset contain 30 images of dimension 3304 × 2336 pixels, 40 images are taken from the DRIVE dataset of size 565 × 584 pixels, 20 images from the STARE dataset of size 700 × 605 pixels, 1200 images from the MESSIDOR dataset of resolution 1440 × 960, 130 images from the DIARETDB0 dataset of size 1500 × 1152 pixels, 89 images from the DIARETDB1 dataset of resolution 1500 × 1152 pixels.

**Figure 8:**
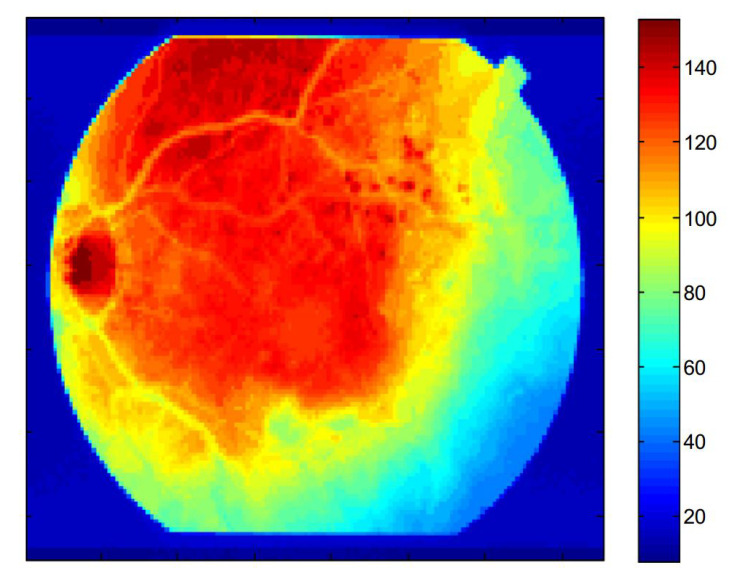
Example of a retinal image with hemorrhages. The color bars show the relative intensity of the image.

The testing dataset is classified into two classes, i.e., healthy images and DR images identified by given datasets specialist ophthalmologists, who split the 1509 images showing hemorrhages into 573 images. When the proposed method was tested on healthy images, no hemorrhages was detected. Table 2 shows a complete description of all used datasets. Figure 8 shows an example of digital retinal imaging with hemorrhages present.

### 4.2. Performance Evaluation Criteria

The results of our proposed method are analysed using several metrics, i.e., accuracy (Acc), sensitivity (Sn), specificity (Sp), Area under Receiver Operating Characteristic (ROC) curve also known as Area Under Curve (AUC), Positive Predicted Value (PPV) and F1 score (F1). These parameters are used to compare the performance of the proposed system with other algorithms. These metrics are defined as follows:

(24) $$Acc=\frac{\left(TP+TN\right)}{\left(TP+FP+TN+FN\right)}\times 100\%,$$

(25) $$Sn=\frac{TP}{\left(TP+FN\right)}\times 100\%,$$

(26) $$Sp=\frac{TN}{\left(TN+FP\right)}\times 100\%,$$

(27) $$PPV=\frac{TP}{\left(TP+FP\right)}\times 100\%,$$

(28) $$F1=2*\frac{Precision*Recall}{Precision+Recall},$$

where TP stand for True Positive values, TN stand for True Negative values, FP stand for False Positive values, and FN stand for False Negative values.

### 4.3. Results and Discussion

The proposed method is performed using different evaluation metrics such as Acc, Sn, Sp, PPV, and F1. A total of 1509 retinal fundus images are used using six different databases. The evaluation of the proposed system is performed for the detection of the hemorrhages. The results are also compared with some existing state-of-the-art methods for each dataset to check superiority and effectiveness. Each experiment is replicated 10 times and their mean results are considered. Table 3 displayed the results of hemorrhage detection using different datasets. HRF and DRIVE mainly have normal subjects and contain good quality images therefore the proposed method showed 100% results. Although using other datasets, the accuracy of our proposed method is still above 95%.

Table 4 displays the quantitative comparison of our proposed system with other state-of-the-art methods and it can perceive that the proposed method outperforms the other algorithms even for a large dataset. By comparison the results obtained by Tang et al. [20] shows better performance than the remaining algorithms as it has the sensitivity of 93%, Tan et al. [33] has the specificity of 96.93% which is even better than the remaining methods, and Qureshi et al. [40] has the highest accuracy of 98% but the proposed system outperforms all the other state-of-the-art algorithms and shows the sensitivity of 97.54%, specificity of 97.89%, and accuracy of 98.22% using HRF, DRIVE, STARE, MESSIDOR, DIARETDB0, and DIARETDB1 datasets. Our proposed system has attained high values of sensitivity, specificity, and accuracy when compared with other methods as highlighted in bold text. The reason for the enhancement is the use of the modified contrast enhancement algorithm, 3D CNN based model for the segmentation, feature extraction using transfer learning, and feature fusion and classification which are not used by other authors. The detection of the hemorrhages is much better than existing methods because of the proper modeling of the hemorrhages rather than just detecting the dark region from the retinal fundus image.

**Figure 9:**
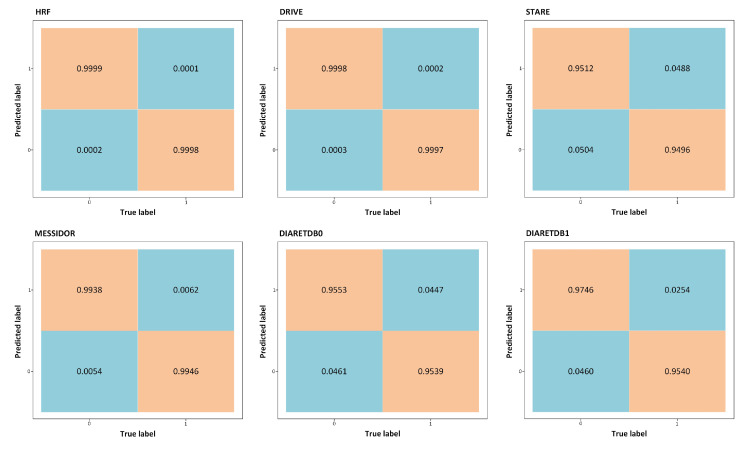
Confusion matrices for retinal HRF, DRIVE, STARE, MESSIDOR, DIARETDB0, and DIARETDB1 datasets.

**Figure 10:**
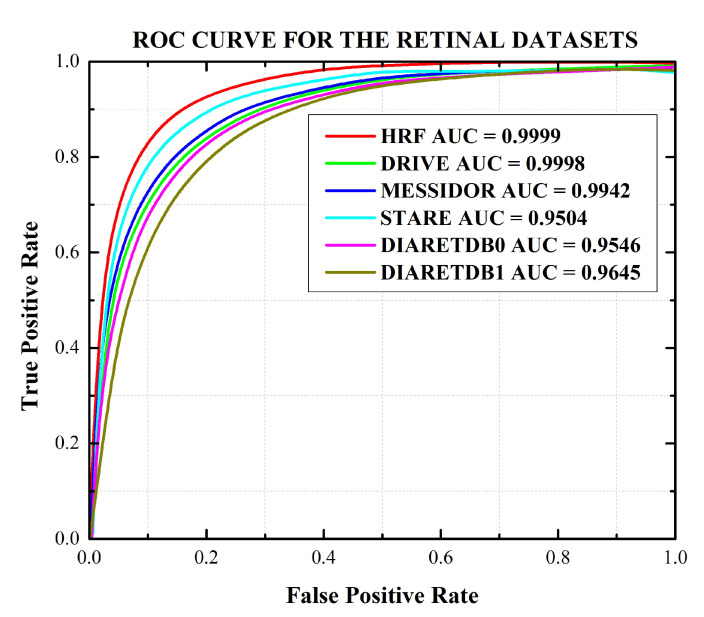
Receiver operating characteristic (ROC) plot for retinal HRF, DRIVE, STARE, MESSIDOR, DIARETDB0, and DIARETDB1 datasets.

The performance of our proposed system is also demonstrated using Confusion Matrix and ROC curves. The confusion matrix of HRF, DRIVE, STARE, MESSIDOR, DIARETDB0, and DIARETDB1 datasets is shown in Figure 9. AUC is also a main quantitative metric that is acquired from ROC curves. The ROC curves plot against the false-positive rates (1-specificity) and true positive rate (sensitivity) by controlling the threshold values of the acquired probability maps which are used to get the hemorrhages. The AUC values are evaluated for the HRF, DRIVE, STARE, MESSIDOR, DIARETDB0, and DIARETDB1 datasets. The ROC curve plot is shown in Figure 10.

Grading results of hemorrhage detection (with statistical 95% confidence intervals) are given in Table 5. The table displayed data from the aforementioned datasets (HRF, DRIVE, STARE, MESSIDOR, DIARETDB0, and DIARETDB1). The proposed system gives PPV, F1 and AUC of 99.99%, 99.98% and 99.99% on HRF, 99.98%, 99.97% and 99.98% on DRIVE, 95.12%, 95.03% and 95.04% on STARE, 99.38%, 99.41%, and 99.42% on MESSIDOR, 95.53%, 95.45% and 95.46% on DIARETDB0, and 97.46%, 96.46% and 96.45% on DIARETDB1 databases, respectively.

### 4.4. Computational Efficiency

Table 6 shows the time execution (in seconds) for each dataset image. Previous methods often have failed to provide the computational efficiency of their proposed approaches. The results displayed in Table 6 reveal that the execution of our proposed method takes 16.78 (s) for HRF, 15.87 (s) for DRIVE, 16.01 (s) for STARE, 17.54 (s) for MESSIDOR, 16.44 (s) for DIARETDB0, and 15.46 (s) for DIARETDB1 databases. However, authors of [20,33] required 18 (s) and 37.5 (s), respectively, to detect the hemorrhages. The study [20] used a computer equipped with a two-core Intel X9650 processor running at 3.00 GHz. The study [33] used a computer with Intel Xeon 2.20 GHz (E5-2650 v4) processor and 512GB RAM. This study used a laptop with an Intel(R) Core(TM) i7−9750H2.6 GHz processor. So the computer equipment was comparable (according to https://www.cpubenchmark.net/ (accessed on 29 May 2021), our computer’s CPU performance is similar to CPU used in [33], but better than CPU used in [20]). Since our main aim is to enhance the visualization to detect the hemorrhages, we will aim to further reduce the execution time in future work.

Overall by comparison the proposed method exhibits improved performance towards the detection of the hemorrhages. The proposed method can be used for real-time evaluation and help the ophthalmologists in automated retinal image analysis.

## 5. Conclusions

Various hemorrhage detection methods have been presented to extract hemorrhage localization that is used to improve the medical analysis of retinal images. However, these methods have numerous shortcomings, such as hemorrhages occurring in image locations where the contrast is poor, false alert because of the existence of blood vessels, and detection performance may vary by disparate sizes of MAs and hemorrhages.

This paper aimed to resolve the aforesaid concerns by the proposed 3D CNN based segmentation model for hemorrhage detection and classification. Firstly, the input retinal image is preprocessed using the modification to the legacy CLAHE method. Then by using the proposed 3D CNN based architecture the hemorrhages are detected from the retinal image and the transfer learning based feature extraction is used to retrain the modified VGG19 based CNN model. Afterwards, the features are selected using the MRCEV algorithm, and the ELM classifier is utilized to detect hemorrhages.

The proposed method was applied to 1509 color fundus images from the six (HRF, DRIVE, STARE, MESSIDOR, DIARETDB0, and DIARETDB1) datasets, and achieved an accuracy of 99.98%, 99.98%, 95.12%, 99.38%, 95.53%, and 97.46% respectively. Moreover our proposed method provides visually pleasant and high-quality results and is more efficient for the automatic detection of the hemorrhages and outperforms other methods. The hemorrhages are detected accurately with less amount of computation time, and the proposed method produces superior results.

In the future, the proposed system will be further analyzed and improved for other application areas of biomedical image processing such as breast cancer and brain tumour detection.

## Figures and Tables

**Table 1:** Proposed 3D-CNN architecture layers.

Layers	Types	Number of Feature Maps	Kernel Size to Form each Feature Map	Stride	Padding
1	Input Layer	3	32 × 32 × 3		
2	Convolutional Layer	16	3 × 3	[1 1]	[1 1 1 1]
3	ReLU				
4	Max Pooling Layer	32	2 × 2	[1 1]	[0 0 0 0]
5	Convolutional Layer	32	3 × 3	[1 1]	[1 1 1 1]
6	ReLU				
7	Convolutional Layer	64	3 × 3	[1 1]	[1 1 1 1]
8	ReLU				
9	Max Pooling Layer	64	2 × 2	[2 2]	[0 0 0 0]
10	Transpose Convolutional Layer	64	4 × 4	[2 2]	
11	Convolutional Layer	128	1 × 1	[1 1]	[0 0 0 0]
12	Softmax Layer				
13	Classification Layer	Cross entropy loss			

**Table 2:** Complete description of database.

Database	Number of Images	Normal	DR
HRF	30	15	15
DRIVE	40	33	7
STARE	20	12	8
MESSIDOR	1200	851	349
DIARETDB0	130	20	110
DIARETDB1	89	5	84
**Total Images**	**1509**	**936**	**573**

**Table 3:** Summary of hemorrhage detection.

Database	Test Images	Correctly Detected	Accuracy (%)
HRF	15	15	100
DRIVE	40	40	100
STARE	20	19	95
MESSIDOR	349	347	99.42
DIARETDB0	110	105	95.45
DIARETDB1	84	81	96.42
**Total**	**618**	**607**	**98.22**

**Table 4:** Performance comparison between our proposed method and other algorithms for diabetic retinopathy detection. N.A—the data is not provided.

Authors	Datasets	Method	Sensitivity	Specificity	Accuracy
Tang et al. [20]	MESSIDOR	Splat feature	93%	66%	-
Mumtaz et al. [32]	DIARETDB1	Scale based	84%	87%	89%
Tan et al. [33]	CLEOPATRA	CNN	62.57%	96.93%	-
Qureshi et al. [40]	EyePACS	ADL-CNN	92.20%	95.10	98%
García et al. [54]	MESSIDOR	Four neural network	86%	-	83.08%
Sinthanayothin et al. [55]	-	Moat operator	77.5%	88.7%	-
Acharya et al. [56]	-	Simple morphological operations	82%	86%	-
Zhang et al. [57]	DIARETDB1	Multi-scale correlation filtering	88.1%	89.3%	90.6%
Saleh et al. [58]	-	Decision support	87.53%	95.08%	-
**Our Proposed Method**	HRF, DRIVE, STARE, MESSIDOR, DIARETDB0, and DIARETDB1	3D CNN	**97.54%**	**97.89%**	**98.22%**

**Table 5:** Hemorrhage detection results with 95% confidence interval (CI).

Database	Sensitivity (%)	Specificity (%)	Accuracy (%)	PPV (%)	F1 Score (%)	AUC (%)
HRF	99.98 (99.96–99.99)	99.98 (95.96–99.99)	99.98 (99.97–99.99)	99.99 (99.98–99.99)	99.98 (99.95–99.99)	99.99 (99.97–99.99)
DRIVE	99.97 (99.96–99.98)	99.97 (99.94–99.98)	99.97 (99.94–99.98)	99.98 (99.96–99.99)	99.97 (99.95–99.98)	99.98 (99.97–99.99)
STARE	94.96 (94.92–94.98)	95.11 (95.07–95.15)	95.04 (95.01–95.07)	95.12 (95.08–95.16)	95.03 (95.00–95.07)	95.04 (95.02–95.06)
MESSIDOR	99.45 (99.42–99.47)	99.38 (99.35–99.41)	99.42 (99.39–99.45)	99.38 (99.36–99.41)	99.41 (99.39–99.43)	99.42 (99.40–99.43)
DIARETDB0	95.39 (95.36–95.42)	95.52 (95.50–95.55)	95.46 (95.43–95.49)	95.53 (95.51–95.55)	95.45 (95.42–95.47)	95.46 (95.43–95.48)
DIARETDB1	95.49 (95.45–95.54)	97.40 (97.44–97.37)	96.43 (96.40–96.46)	97.46 (97.44–97.49)	96.46 (96.49–96.43)	96.45 (96.42–96.47)

**Table 6:** Computational time for hemorrhage detection.

	HRF	DRIVE	STARE	MESSIDOR	DIARETDB0	DIARETDB1
Time (in seconds)	16.78	15.87	16.01	17.54	16.44	15.46

## Data Availability

The data and program codes are available from the corresponding author upon reasonable request.
